# The Impact of Radiotherapy on the Incidence of Secondary Malignancies: A Pan-Cancer Study in the US SEER Cancer Registries

**DOI:** 10.3390/curroncol28010035

**Published:** 2021-01-08

**Authors:** Wei Li, Haitao Xiao, Xuewen Xu, Yange Zhang

**Affiliations:** Department of Plastic and Burns Surgery, Sichuan University, Chengdu 610041, China; 2017324020055@stu.scu.edu.cn (W.L.); Xiaohaitaoplastic@126.com (H.X.); xuxuewenplastic@126.com (X.X.)

**Keywords:** radiotherapy, first primary malignancies, second primary malignancies, pan-cancer

## Abstract

The population of cancer patients with second primary malignancies (SPMs) is rapidly growing. The relationship between radiotherapy and SPMs for some types of tumors is unknown or debated. In this study, we identify 24 types of first primary malignancies (FPMs) between 2004 and 2015 in the Surveillance, Epidemiology, and End Results (SEER) database. Patients in the radiotherapy group were matched to those in the no radiotherapy group with a matching ratio of 1:1. After propensity-score matching (PSM), additional competing risk regression analyses were performed to calculate the efficacy of radiotherapy to SPMs in the PSM-adjusted population. In addition, the Fine and Gray model was utilized in the primary cohorts, and stratified analyses were performed based on surgery. This study includes a total of 2,831,789 eligible patients with tumors diagnosed from 2004 to 2015 in the SEER 18 database, amongst whom 100,194 (3.5%) patients developed SPMs. We observe higher risks of SPMs associated with radiotherapy in several types of tumors in the PSM-adjusted populations (small bowel adenocarcinoma, small cell lung carcinoma, prostate adenocarcinoma, urinary bladder transitional cell carcinoma, invasive ductal breast carcinoma, invasive lobular breast carcinoma, and Hodgkin lymphoma). The results in the PSM-adjusted populations were consistent with outcomes in the multivariable competing risk models. Meanwhile, in subgroup analyses stratified by surgery, some other types of tumor (except for those with positive results in the PSM-adjusted cohorts) with radiotherapy were also associated with a higher prevalence of SPMs in the subgroups of surgical treatment (pancreatic adenocarcinoma, rectal adenocarcinoma, lung adenocarcinoma and follicular thyroid carcinoma in the surgery subgroups). The impact of radiotherapy on the incidence of secondary malignancies is distinct in different types of cancer. These findings merit further investigation and may ultimately impact treatment decision-making for tumor management.

## 1. Introduction

Radiotherapy remains one of the major treatment modalities for cancers. More than 50% of all cancer patients need radiotherapy at some point in the treatment process [1]. Though radiotherapy can significantly improve the rate of local tumor control, its effect on overall long-term survival is controversial in some types of tumors. One of the major concerns of radiotherapy is that patients with tumors receiving radiotherapy may have higher risks of developing secondary primary malignancies (SPMs) [2,3,4,5,6,7].

Compared with the general population, approximately 14% of patients diagnosed with a common cancer developed a secondary cancer [8]. Except for some genetic and environmental risk factors, treatment methods were also associated with the occurrence of secondary malignancies [1]. Though controversies still existed, some previous studies have observed independent associations of radiotherapy and SPMs in some tumor types, including tumors originated from the prostate, [6,9,10] breast, [7,11,12,13] rectum [14], and uterus [15,16]. However, previous publications have not involved some common cancer types, such as tumors from the esophagus, stomach, and urinary bladder. Gonzalez et al. have performed a comprehensive and representative study related to the impact of radiotherapy on secondary cancers [8]. However, they failed to include all the tumor sites of the prior tumors, and they classified patients by tumor sites instead of pathological types. This study also did not utilize competing risk regression models (such as the Fine and Gray model) considering death and secondary malignancies as competing risks. In addition, they did not include patients after 2002, and some radiotherapy techniques, such as intensity-modulated radiotherapy (IMRT), have not been commonly used at that time [17].

Owing to the shortages and controversies of previous studies, in this study, utilizing the Surveillance, Epidemiology, and End Results (SEER) database, a newest pan-cancer study was carried out to explore the impact of radiotherapy on the occurrence of secondary malignancies.

## 2. Material and Methods

Data from the SEER 18 registry database for 2004 through 2015 was extracted for the present study. The SEER program collects data regarding cancer incidence, patient demographics, tumor parameters, patient treatment, and prognosis from 18 population-based cancer registries, covering approximately 28% of the US population (seer.cancer.gov/about/overview.html). Primary cancer site and histology were coded based on the third edition of the International Classification of Diseases for Oncology (ICD-O-3) in SEER 18. We identified 2,831,789 patients with 24 types of first primary malignancies (FPMs) according to ICD-O-3 site code and histologic code from the SEER database (Supplementary Table S1). We excluded patients without histologic diagnosis and patients without complete survival data and follow-up information. In addition, SPMs diagnosed within one year of the FPMs were excluded to eliminate what were probably multiple primary cancers. SPMs with tumor sites identical to those of their corresponding FPMs were excluded. Additionally, SPMs with histologic features similar to their corresponding FPMs were examined carefully to confirm that the recurrent tumors of FPMs would not be considered as SPMs. The 24 types of FPMs included esophageal adenocarcinoma, esophageal squamous cell carcinoma, gastric adenocarcinoma, hepatocellular carcinoma, intrahepatic cholangiocarcinoma, pancreatic adenocarcinoma, small bowel adenocarcinoma, colon adenocarcinoma (divided into six subgroups [appendix, cecum, ascending colon, transverse colon, descending colon, and sigmoid colon] based on tumor site), rectal adenocarcinoma, lung adenocarcinoma, lung squamous cell carcinoma, small cell lung carcinoma, large cell lung carcinoma, renal cell carcinoma, prostate adenocarcinoma, urinary bladder transitional cell carcinoma, papillary thyroid carcinoma, follicular thyroid carcinoma, invasive ductal breast carcinoma, invasive lobular breast carcinoma, ovarian epithelial carcinoma, Hodgkin lymphoma (nodal), non-Hodgkin lymphoma (nodal) and melanoma (skin).

We extracted data of demographic and clinicopathological characteristics from SEER, including gender, age, race, tumor grade (differentiation), tumor location, American Joint Committee on Cancer (AJCC) stage, chemotherapy, radiotherapy, and surgery. Race was divided into three groups (white, black, and others). Tumor grade was divided into four groups: Well-differentiated (grade I), moderately-differentiated (grade II), poorly-differentiated (grade III), and un-differentiated (grade IV). The Tumor Node Metastasis (TNM) stage was based on the AJCC (6th edition) staging system. SPMs were treated as time-to-event data, and the time to an SPM was calculated as the period between the date of diagnosis of the FPMs and the date of the diagnosis of the SPMs.

### Statistical Analysis

Patients were divided into two groups based on whether they had received radiotherapy as part of their cancer treatment or not. The radiotherapy techniques included external beam, brachytherapy, or a combination of external beam and brachytherapy. Most of the patients with thyroid cancer received radioisotopes, while not the external beam radiation (Table 1). To explore the association of radiotherapy with the incidence of SPMs, competing-risks regression models (the Fine and Gray method) were utilized in the original cohorts. In this method, death and SPMs were considered as competing events. In the competing risk regression models, variables, including gender, age, race, tumor differentiation (grade), were adjusted in model 1 and variables, including gender, age, race, tumor differentiation (grade), AJCC-TNM stage, surgery, and chemotherapy (if available), were adjusted in model 2. Owing to the limitations of Fine and Gray models, the proportional sub-distribution hazard assumption in the competing risk model is often impossible to hold over a long time of follow-up [18], thus in some multivariable models, Cox models were utilized to calculate the effect sizes of radiotherapy vs. no radiotherapy. In Cox models, SPMs were deemed as the only event.

Propensity scores were used to minimize the effect of bias caused by differences in clinicopathologic parameters between the two groups. Propensity scores were estimated according to the following variables: Gender, age, race, tumor differentiation (grade), tumor location, AJCC-TNM stage, surgery, and chemotherapy. With a matching ratio of 1:1, patients in the radiotherapy group were matched to those in the no radiotherapy group with the closest estimated propensity score within 0.02 of the standard deviation of the logit-transformed propensity score. After propensity-score matching (PSM), the variables of the PSM cohorts were then compared to confirm that the baseline features were well-balanced between the two groups. Based on the cohorts produced by PSM methods, an additional competing risk regression analysis was performed to calculate the efficacy of radiotherapy on incidences of SPMs.

A *p* value less than 0.05 was deemed statistically significant. All statistical analyses were performed by R (http://www.R-project.org) and EmpowerStats software 2.0 (www.empowerstats.com, X&Y solutions, Inc. Boston, MA, USA).

## 3. Results

### 3.1. Patient Characteristics

This study enrolled a total of 2,831,789 patients meeting the inclusion criteria with tumors diagnosed from 2004 to 2015 in the SEER 18 registry, amongst whom 100,194 (3.5%) patients developed a second primary malignancy. The proportions of FPMs and SPMs with radiotherapy in terms of the 24 cancer types (based on tumor sites and histologic types) were shown in Table 1. Patients with follicular thyroid carcinoma (54.9%), papillary thyroid carcinoma (48.8%), invasive ductal breast carcinoma (48.4%), invasive lobular carcinoma (44.6%), rectal adenocarcinoma (42.0%), and esophageal adenocarcinoma (21.0%) had the highest radiotherapy rates among patients with FPMs. In patients with SPMs, these five tumor types still had the highest radiotherapy rates (Table 1). In the total cohort, the date of FPMs to the date of the newly diagnosed SPMs was distinct across different types of tumor (median range from 29 to 60 months, mean from 38.8 to 63.7 months).

**Figure 1:**
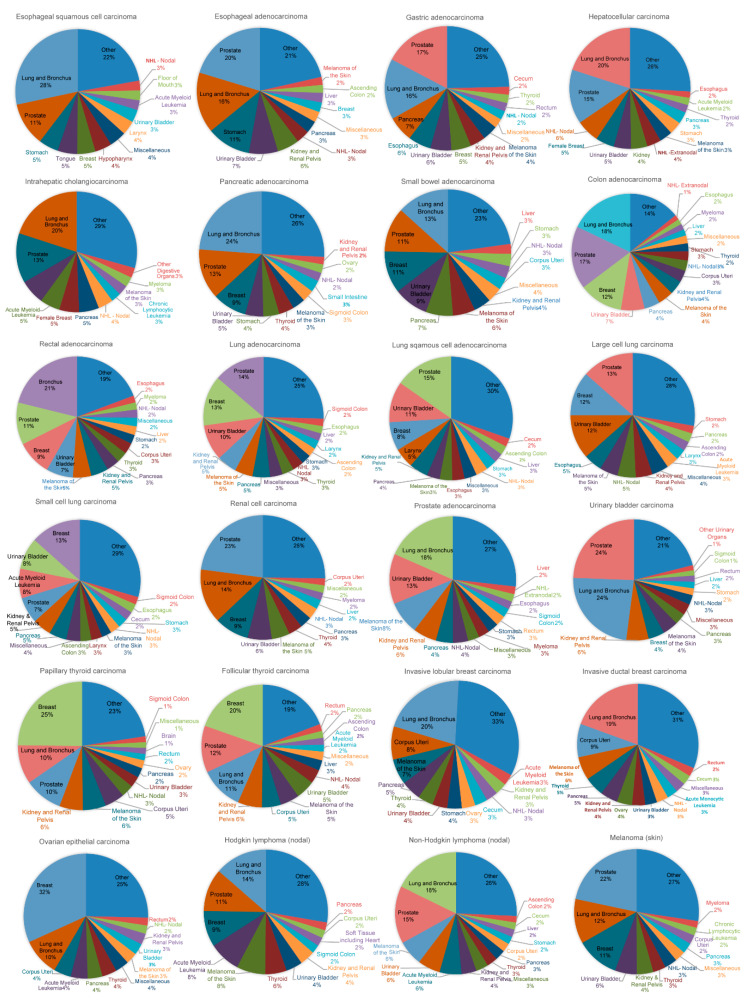
The proportion of the sites of the second primary malignancies of the 24 types of primary cancers.

Figure 1 displays the patterns of SPMs (proportions of the tumor sites) based on the primary pathological tumor types. The patterns of adenocarcinoma and squamous carcinoma were different. The five most common sites of SPMs were lung and bronchus (28%), prostate (11%), stomach (5%), tongue (5%) and breast (5%) for esophageal squamous carcinoma, and prostate (20%), lung and bronchus (16%), stomach (11%), urinary bladder (7%) and kidney (6%) for esophageal adenocarcinoma, respectively. The most common types of SPMs were also different among patients with lung squamous cell carcinoma, lung adenocarcinoma, large cell lung carcinoma, and small cell lung carcinoma (shown in Figure 1 in detail). Notably, the most common types and prevalence of SPMs for invasive ductal breast carcinoma and invasive lobular carcinoma, and for follicular thyroid carcinoma and papillary thyroid carcinoma were similar. The patterns of SPMs were similar for patients with small bowel adenocarcinoma and colorectal adenocarcinoma (the three most common sites of SPMs were lung and bronchus, prostate and breast for the two types of tumor). The proportions of SPMs for the other FPMs were shown in Figure 1 in detail.

### 3.2. Association between Radiotherapy and Incidence of SPMs in Non-Adjusted Competing Risk Models

**Figure 2:**
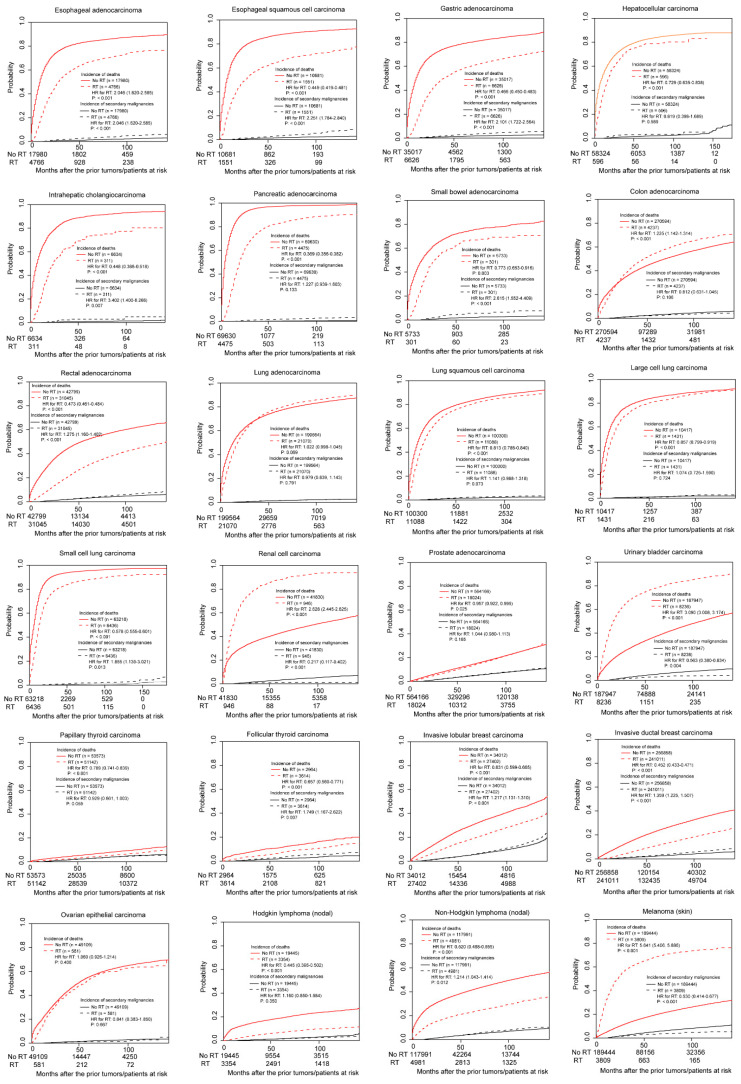
The cumulative incidences of death and secondary primary malignancy in the non-adjusted primary cohorts.

Figure 2 shows the results of the non-adjusted Fine and Gray models. Radiotherapy was associated with more SPMs in patients with esophageal adenocarcinoma, esophageal squamous cell carcinoma, gastric adenocarcinoma, intrahepatic cholangiocarcinoma, small bowel adenocarcinoma, rectal adenocarcinoma, small cell lung carcinoma, follicular thyroid carcinoma, invasive lobular breast carcinoma, invasive ductal breast carcinoma, and non-Hodgkin lymphoma (all *p* < 0.05). In contrast, radiotherapy was related to fewer SPMs in patients with renal cell carcinoma, urinary bladder transitional cell carcinoma, and skin melanoma (all *p* < 0.05). No associations between radiotherapy and SPMs were observed in the other tumor types, including hepatocellular carcinoma, pancreatic adenocarcinoma, colon adenocarcinoma (Supplementary Figure S1), lung adenocarcinoma, lung squamous cell carcinoma, large cell lung carcinoma, prostate adenocarcinoma, papillary thyroid carcinoma, ovarian epithelial carcinoma, and Hodgkin lymphoma (all *p* > 0.05).

### 3.3. Association between Radiotherapy and Incidence of SPMs in PSM Cohorts

**Figure 3:**
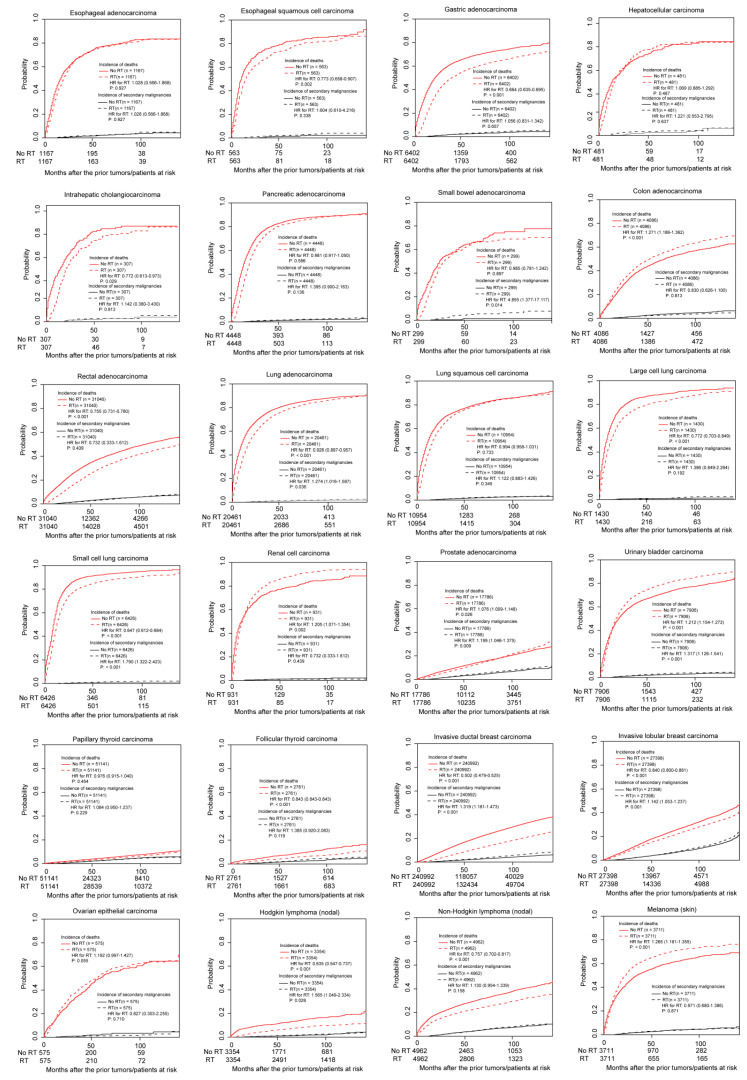
The cumulative incidences of death and secondary primary malignancy in the PSM-adjusted populations.

The impact of radiotherapy on the incidence of SPMs was different across cancers. In the PSM-adjusted population, according to the outcomes of the survival analysis (Kaplan-Meier) considering the competing-risks in event occurrence, the results could be divided into two groups: (I) Patients receiving radiotherapy were associated with more SPMs and (II) Patients who received radiotherapy showed similar SPM incidences. Group I included the following tumor types: Small bowel adenocarcinoma (*p* = 0.014), small cell lung carcinoma (<0.001), prostate adenocarcinoma (*p* = 0.009), urinary bladder transitional cell carcinoma (*p* < 0.001), invasive ductal breast carcinoma (*p* < 0.001), invasive lobular breast carcinoma (*p* = 0.001) and Hodgkin lymphoma (nodal; *p* = 0.028) (Table 2). The other tumor types were assigned to Group II, due to the similar SPM incidences for patients receiving or not receiving radiotherapy. Notably, in supplementary Figure S1, there were still no associations between radiotherapy and SPMs for patients with colon adenocarcinoma stratified by tumor site (cecum, appendix, ascending colon, transverse colon, descending colon, and sigmoid colon). Figure 3 displays the cumulative incidences of death and secondary malignancy of the 24 types of tumor in the PSM cohorts.

### 3.4. Association between Radiotherapy and Incidence of SPMs in Multivariable Competing Risk Models

As shown in Table 3, in the primary cohorts, we adjusted different variables in two models. Model II was also adjusted for tumor stage and treatment (surgery and chemotherapy) except for variables in model I (age, sex, race, and tumor grade). The effect and significance in most of the results in model 2 were consistent with the results in the PSM-adjusted population. Patients with small bowel adenocarcinoma (*p* = 0.004), small cell lung carcinoma (*p* = 0.043), prostate adenocarcinoma (*p* < 0.001), urinary bladder transitional cell carcinoma (*p* < 0.001), and invasive lobular carcinoma (*p* < 0.001) still had higher incidence of SPMs after radiotherapy. However, due to the data limitation (failed to meet the proportional distribution hazard assumption in both Cox and Fine and Gray models), there were no results for patients with invasive ductal breast carcinoma after adjusting the confounding factors. In addition, in the primary cohorts, a higher prevalence of SPMs was observed in patients with pancreatic adenocarcinoma after radiotherapy (*p* = 0.019). In both models, there was no significant difference in SPM rate for Hodgkin lymphoma (nodal) after radiotherapy or not (*p* = 0.351). Notably, for patients with several types of tumor (esophageal tumors, gastric adenocarcinoma, intrahepatic cholangiocarcinoma, ascending colon adenocarcinoma, lung adenocarcinoma, lung squamous cell carcinoma, renal cell carcinoma, non-Hodgkin lymphoma (nodal) and melanoma), the significant differences in model I were not observed in model II after adjusting additional covariates, including treatment and tumor stage. 

### 3.5. Stratified Analyses

In supplementary Table S2, we performed subgroup analyses based on surgical treatment for patients whose major treatment method was surgical resection (exclude lymphoma and small cell lung carcinoma). In the surgery subgroups, the small bowel adenocarcinoma (*p* = 0.013), prostate adenocarcinoma (*p* < 0.001), urinary bladder transitional cell carcinoma (*p* < 0.001), and invasive lobular breast carcinoma (*p* < 0.001) still showed consistent effect and significance with the previous multivariable and PSM-adjusted cohort analyses. However, in the no-surgery subgroups, owing to the sparse data (limited number of patients with SPMs), no reasonable results related to these tumor types were yielded by the multivariable models.

In the surgery subgroups, several types of tumor with no significance in the PSM-adjusted population became significant (Supplementary Table S2). These types of tumor included pancreatic adenocarcinoma (*p* = 0.023), rectal adenocarcinoma (*p* < 0.001), lung adenocarcinoma (*p* = 0.003) and follicular thyroid carcinoma (*p* = 0.010) in the surgery subgroups. More SPMs were observed after radiotherapy for patients with these types of tumor.

## 4. Discussion

Radiotherapy has been deemed as a double-edged sword, since it is a well-established treatment modality for solid cancers, but is simultaneously likely to induce new primary cancers years after the radiation in a small number of patients. In this study, we analyzed the effect of radiotherapy on the incidence of SPMs in 24 cancer types. In the PSM-adjusted population, several tumor types who underwent radiotherapy showed a higher prevalence of SPMs. These types of tumor include small bowel adenocarcinoma, small cell lung carcinoma, prostate adenocarcinoma, urinary bladder transitional cell carcinoma, invasive ductal breast carcinoma, invasive lobular breast carcinoma, and Hodgkin lymphoma. In the surgical treatment subgroup, the effect of several other types of tumor (pancreatic adenocarcinoma, rectal adenocarcinoma, lung adenocarcinoma, and follicular thyroid carcinoma) became significant in the multivariable models (Supplementary Table S2). All these types of tumors should be with caution when carrying out radiotherapy, and the radiological doses should be carefully managed.

We performed stratified analyses based on surgery because patients receiving surgical treatment represented a specific population, usually with more favorable performance status and earlier tumor stage [8,14,19]. In addition, patients undergoing surgery usually had a longer survival compared to those without surgery, thus more patients in the surgery subgroup had SPMs during the follow-up. Consequently, the impact of radiotherapy on the incidence of SPMs for patients with or without surgery may be different. Actually, patients with combined surgery and radiotherapy can achieve a higher local tumor control rate and better long-term survival. Surgery and radiotherapy were often performed simultaneously in many types of tumors [20,21,22,23]. In other words, most of the patients without surgery also had not received radiotherapy, thus, we cannot yield a reasonable effect of radiotherapy based on the sparse data (few patients received radiotherapy) in the no surgery subgroup in some types of tumor (Supplementary Table S2).

There were several advantages of this study compared to previously published literature on this topic. Firstly, we explored the effect of radiotherapy on SPMs based on the pathological types of FPMs in corresponding tumor sites. It is more reasonable than previous studies, which only utilized sites to classify tumor types. Patients with different pathological types may have different responses to radiotherapy (e.g., small and non-small lung carcinoma; adenocarcinoma and squamous cell carcinoma) [24,25]. Secondly, this study is a systematic and comprehensive study of many types of tumors that were received radiotherapy with data from the SEER database, thus we can compare risks across first tumor types and evaluate common patterns of risk. In contrast, previous studies did not explore the overall effect of radiotherapy on SPMs. Most of the publications, including several studies of the SEER database, focused on a single FPM or a single SPM [3,6,7,9,13,15,19,23,26,27,28]. Third, the strength of this study lies in that the outcomes were confirmed by adequate statistical analyses in large population-based cohorts. PSM analysis considering competing risk allowed balancing of many covariates (e.g., age, sex, race, tumor grade, and stage) without the common statistical concerns for regression analysis. To avoid selection bias of PSM methods, we also utilized Fine and Gray models in the primary cohorts, adjusting the confounding factors. Additionally, we conducted a stratified analysis by surgery to validate the conclusions.

Previous studies have demonstrated higher risks of SPMs for patients with breast tumors, prostate tumors, and Hodgkin lymphoma receiving radiotherapy [1,8]. The conclusions in this study were consistent with the literature. However, some inconsistent phenomena were also observed in the present study. An increase in the prevalence of SPMs would be the most important late effect that could occur in patients with thyroid cancers treated with radioisotopes [29,30]. However, in this study, we did not observe a higher risk for SPMs associated with radioisotopes (in both PSM and multivariable models) in patients with papillary thyroid carcinoma. The possible reasons for this discrepancy were the different sample size and different follow-up of the study populations. Additionally, in future studies, maybe it is necessary to provide physicians with quantitative information on the risk of developing SPMs after radioisotope treatment, which will make the decision to treat a patient with radioisotopes incorporating a careful risk-benefit analysis. Except for thyroid tumors, a previous study by Warschkow et al. demonstrated that the risk of SPMs was slightly decreased after radiation in patients with rectal adenocarcinoma after resection [14]. The opposite conclusion in this study (increased risk of SPMs after radiotherapy in patients with rectal adenocarcinoma after surgery) may be caused by the increased use of IMRT in recent years, [31,32,33] which may increase the chance of SPMs. The study of Warschkow et al. have included patients in the era before IMRT was widely used. For patients with other types of tumors, there were only a few existed publications related to this topic. In the present study, for the first time, we presented the newest landscape for the impact of radiotherapy on SPMs across 24 cancer types, which is significant to guide the clinical decision-making for managing tumors.

Notably, for different tumor types, the role of radiation was distinct. For example, surgical resection was the predominant treatment method for early-stage prostate, bladder, and breast cancers, and radiotherapy was only the adjuvant therapy for some late-stage patients with these types of tumor. However, for small cell lung cancer, [34] radiotherapy played an important role in managing cases with both limited and extensive stages of tumor. Consequently, though radiotherapy had a significant impact on the incidence of SPMs in all of the above tumor types, the instructional clinical values were different. For surgery-based tumors, early-stage prostate, bladder, and breast cancers, radiotherapy should be carried out with caution, and informed consent should be obtained. In contrast, for small cell lung cancer, radiotherapy should be used as one of the major treatment regimens, and the optimal doses and courses of treatment should be explored in future studies.

Admittedly, there are several limitations to this study. Like other observational researches, the main drawback of the data from SEER is the lack of treatment randomization, which may bias our results. We utilized both PSM and multivariable competing risk models to adjust confounders. And the stratified analysis based on surgery was performed to minimize potential bias. In addition, we did not have information on the doses and modalities of radiotherapy, which may impact the second cancer risk. However, in this study, we included patients after 2004, and some techniques, including IMRT, was introduced and commonly used. The second limitation for this study is that we used two types of models for multivariable analysis, due to the sparse data. Actually, the results from both Cox and Fine and Gray models were similar in cancer types that both methods were available (data are not shown). In the PSM-adjusted population, Fine and Gray models can be used in all the tumor types, which made the results more reliable and explicable. The third limitation lies in that we failed to perform more subgroup analyses based on other potential interactive factors, including age of diagnosis, time to the prior cancer, and AJCC stage. More studies were needed to illustrate the effect and significance of radiotherapy among some specific populations. Regarding fourth limitation, we demonstrated a higher risk of SPMs of patients with small cell lung carcinoma and small bowel adenocarcinoma after radiotherapy. Actually, for patients with these types of tumor, only a small proportion of patients have received radiotherapy in the SEER database, thus, the conclusions related to the benefit and long-term side effect of radiotherapy for these types of tumors should also be validated in further studies. Finally, given the difficulty to identify the regional organs that were influenced by the radiations, the total number of SPMs after radiotherapy was used as the endpoint in this study, which may have an impact on the conclusions. However, emerging studies indicated that the biological effects of radiotherapy were not limited to targeted regions. As a result of signal transmission from an irradiated cell, a plethora of biological phenomena occurred in non-irradiated cells (the radiation-induced bystander effect). The mechanisms underlying it should be totally illustrated in further studies. Based on this theory, in this study, we still used the total number of SPMs as the observed endpoint.

## 5. Conclusions

We present a comprehensive and newest pan-cancer study illustrating the effect of radiotherapy on the incidence of SPMs. To our knowledge, this is the first pan-cancer study exploring the role of radiotherapy in different pathological types of tumors. We have observed higher risks of SPMs in several types of tumors in the PSM-adjusted populations. Meanwhile, in subgroup analyses stratified by surgery, some types of tumor were also associated with the prevalence of SPMs in the surgery subgroups except for those with positive results in the PSM-adjusted cohorts. All these findings were clinically important for the selection of radiotherapy in managing cancers. However, further studies are still needed to validate the conclusions in the present study.

## Figures and Tables

**Table 1:** Patient characteristics.

Prior Cancer	Number of Patients with FPMs			Number of Patients with SPMs			Time to a Secondary Malignancy		
	Total Number	Radiation *	No Radiation *	Total Number	Radiation *	No Radiati on *	Total Cohort **	Radiation **	No Radiation **
Esophagus									
Adenocarcinoma	22,746	4766 (21.0)	17,980 (79.0)	390	137 (35.1)	253 (64.9)	47.6 (40.5)	44.8 (37.0)	49.1 (42.0)
Squamous cell carcinoma	12,232	1551 (12.7)	10,681 (87.3)	230	58 (25.2)	172 (74.8)	45.9 (37.0)	53.9 (47.5)	43.2 (36.0)
Gastric adenocarcinoma	41,643	6626 (15.9)	35,017 (84.1)	719	204 (28.4)	515 (71.6)	43.7 (36.0)	45.6 (40.5)	42.9 (34.0)
Liver									
Hepatocellular carcinoma	58,920	596 (1.0)	58,324 (99.0)	987	16 (1.6)	971 (98.4)	46.0 (34.0)	28.7 (20.0)	46.3 (34.0)
Intrahepatic cholangiocarcinoma	6945	311 (4.5)	6634 (95.5)	51	7 (13.7)	44 (86.3)	42.9 (34.0)	38.1 (29.0)	43.6 (35.5)
Pancreatic adenocarcinoma	74,105	4475 (6.0)	69,630 (94.0)	244	81 (33.2)	163 (66.8)	38.8 (29.0)	43.1 (31.0)	36.6 (28.0)
Small bowel adenocarcinoma	6034	301 (5.0)	5733 (95.0)	117	14 (12.0)	103 (88.0)	43.0 (36.0)	42.7 (34.5)	43.0 (36.0)
Colon adenocarcinoma									
Cecum	62,641	827 (1.3)	61,594 (98.7)	2006	26 (1.3)	1980 (98.7)	47.7 (41.0)	39.2 (22.0)	47.9 (42.0)
Appendix	6382	118 (1.8)	6264 (98.2)	106	6 (5.7)	102 (94.3)	43.3 (35.5)	30.2 (27.5)	43.8 (35.5)
Ascending colon	54,343	456 (0.8)	53,887 (99.2)	1967	8 (0.4)	1959 (99.6)	48.5 (41.0)	60.2 (36.2)	48.5 (41.0)
Transverse colon	26,637	189 (0.7)	26,448 (99.3)	901	6 (0.7)	895 (99.3)	48.2 (40.0)	64.7 (32.1)	48.1 (40.0)
Descending colon	16,721	225 (1.3)	16,496 (98.7)	526	4 (0.8)	522 (99.2)	50.4 (43.0)	51.0 (45.0)	50.4 (53.0)
Sigmoid colon	74,642	2099 (2.8)	72,543 (97.2)	2567	59 (2.3)	2508 (97.7)	50.4 (44.0)	48.5 (38.0)	50.4 (44.0)
Rectal adenocarcinoma	73,835	31,045 (42.0)	42,790 (58.0)	2495	1183 (47.4)	1312 (52.6)	50.6 (45.0)	52.9 (47.0)	48.5 (42.0)
Lung									
Adenocarcinoma	220,634	21,070 (9.5)	199,564 (90.5)	3046	182 (6.0)	2764 (94.0)	44.6 (37.0)	41.3 (32.5)	44.9 (38.0)
Squamous cell carcinoma	111,388	11,088 (10.0)	100,300 (90.0)	1669	211 (12.6)	1458 (87.4)	41.6 (35.0)	41.3 (36.0)	41.6 (35.0)
Small cell carcinoma	69,654	6436 (9.2)	63,218 (90.8)	332	70 (21.1)	262 (78.9)	52.3 (42.0)	51.7 (41.0)	52.4 (42.0)
Large cell carcinoma	11,848	1431 (12.1)	10,417 (87.9)	155	23 (14.8)	132 (85.2)	48.6 (45.0)	65.4 (75.0)	45.6 (42.0)
Renal cell carcinoma	42,776	946 (2.2)	41,830 (97.8)	1474	8 (0.5)	1466 (99.5)	51.1 (45.0)	31.6 (28.0)	51.2 (45.0)
Prostate adenocarcinoma	582,190	18,024 (3.1)	564,166 (96.9)	30,836	976 (3.2)	29860 (96.8)	62.2 (59.0)	61.7 (58.0)	62.2 (59.0)
Urinary bladder transitional cell carcinoma	196,183	8236 (4.2)	187,947 (95.8)	9969	214 (2.1)	9755 (97.9)	46.5 (39.0)	38.8 (34.5)	46.7 (40.0)
Thyroid									
Papillary thyroid carcinoma	104,715	51,142 ^†^ (48.8)	53,573 (51.2)	2626	1342 (51.1)	1284 (48.9)	48.6 (43.0)	48.8 (44.0)	48.4 (43.0)
Follicular thyroid carcinoma	6578	3614 ^‡^ (54.9)	2964 (45.1)	191	129 (67.5)	62 (32.5)	51.6 (47.0)	50.9 (47.0)	53.0 (47.5)
Breast									
Invasive ductal carcinoma	497,869	241,011 (48.4)	286,858 (51.6)	15,236	8749 (57.4)	6487 (42.6)	55.0 (50.0)	56.4 (52.0)	53.0 (47.0)
Invasive lobular carcinoma	61,414	27,402 (44.6)	34,012 (55.4)	4450	2139 (48.1)	2311 (51.9)	63.7 (60.0)	66.9 (64.0)	60.8 (55.0)
Ovarian epithelial carcinoma	49,690	581 (1.2)	49,109 (98.8)	932	10 (1.1)	922 (98.9)	46.9 (40.0)	58.9 (43.0)	46.8 (40.0)
Lymphoma									
Hodgkin lymphoma (nodal)	22,799	3354 (14.7)	19,445 (85.3)	455	75 (16.5)	380 (83.5)	55.7 (51.0)	56.8 (52.0)	55.5 (50.5)
Non-Hodgkin lymphoma (nodal)	122,972	4981 (4.1)	117,991 (95.9)	5652	320 (5.7)	5332 (94.3)	49.9 (43.0)	55.7 (53.0)	49.6 (43.0)
Melanoma (skin)	193,253	3809 (2.0)	189,444 (98.0)	9865	109 (1.1)	9756 (98.9)	47.6 (40.0)	38.4 (31.0)	47.7 (41.0)

* The number and proportion of patients with or without radiation. ** The mean (median) time of prior cancer to newly diagnosed cancer (months). ^†^ Radioisotopes: 95.5% (*n* = 48,820); Beam radiation: 2.1% (*n* = 1059). ^‡^ Radioisotopes: 93.7% (*n* = 3388); Beam radiation: 3.5% (*n* = 125). FPMs, first primary malignancies; SPMs, second primary malignancies.

**Table 2:** The risk for secondary malignancies after radiation in pan-cancers after PSM.

Prior Cancer	Fine and Gray Models in PSM Cohorts			
	Number	Events	HR (95%CI)	*p* Values
Esophagus				
Adenocarcinoma	2334	57	1.028 (0.566–1.868)	0.927
Squamous cell carcinoma	1126	21	1.604 (0.610–4.216)	0.338
Gastric adenocarcinoma	12,804	371	1.056 (0.831–1.342)	0.657
Hepatocellular carcinoma	962	24	1.221 (0.553–2.795)	0.637
Intrahepatic cholangiocarcinoma	614	13	1.142 (0.380–3.430)	0.813
Pancreatic adenocarcinoma	8896	134	1.395 (0.900–2.163)	0.136
Small bowel adenocarcinoma	598	17	4.855 (1.377–17.117)	0.014
Colon adenocarcinoma				
Cecum	1562	47	1.507 (0.944–2.406)	0.086
Appendix	214	6	1.878 (0.333–10.582)	0.475
Ascending colon	852	17	0.660 (0.248–1.758)	0.406
Transverse colon	362	19	0.478 (0.177–1.293)	0.146
Descending colon	422	10	0.652 (0.175–2.428)	0.523
Sigmoid colon	3890	99	1.209 (0.782–1.870)	0.393
Rectal adenocarcinoma	62,080	2404	0.984 (0.904–1.071)	0.706
Lung				
Adenocarcinoma	40,922	452	1.179 (0.933–1.490)	0.169
Squamous carcinoma	21,908	391	1.122 (0.883–1.426)	0.345
Small cell carcinoma	12,852	109	1.790 (1.322–2.423)	<0.001
Large cell carcinoma	2860	37	1.386 (0.849–2.264)	0.192
Renal cell carcinoma	1862	20	0.732 (0.333–1.612)	0.439
Prostate adenocarcinoma	35572	1783	1.199 (1.046–1.375)	0.009
Urinary bladder transitional cell carcinoma	15,812	405	1.317 (1.126–1.541)	<0.001
Thyroid				
Papillary thyroid carcinoma	102,282	2591	1.084 (0.950–1.237)	0.229
Follicular thyroid carcinoma	5522	140	1.385 (0.920–2.083)	0.119
Breast				
Invasive ductal carcinoma	481,984	15070	1.319 (1.181–1.473)	<0.001
Invasive lobular carcinoma	54,796	4074	1.142 (1.053–1.237)	0.001
Ovarian epithelial cancer	1150	24	0.827 (0.303–2.255)	0.710
Lymphoma				
Hodgkin lymphoma (nodal)	6708	124	1.565 (1.049–2.334)	0.028
Non-Hodgkin lymphoma (nodal)	9924	608	1.130 (0.954–1.339)	0.158
Melanoma (skin)	7422	228	0.971 (0.680–1.386)	0.871

Propensity score matching was based on age, sex, race, tumor grade, American Joint Committee on Cancer (AJCC) stage, chemotherapy, surgery, and year of diagnosis if available.

**Table 3:** The risk for secondary malignancies after radiation in multivariable models.

Prior Cancer	Fine and Gray Models							
	Model I				Model II			
	Number	Events	HR (95%CI)	*p* Value	Number	Events	HR (95%CI)	*p* Value
Esophagus								
Adenocarcinoma	22,746	390	1.895 (1.516–2.369)	<0.001	22,650	388	0.856 (0.524–1.399)	0.535
Squamous cell carcinoma	12,232	230	2.085 (1.668–2.607)	<0.001	12,194	230	0.694 (0.458–1.053)	0.086
Gastric adenocarcinoma	41,643	719	1.898 (1.547–2.329)	<0.001	41,523	718	0.954 (0.764–1.190)	0.674
Hepatocellular carcinoma	58,920	987	0.494 (0.186–1.316)	0.159	48,292	674	1.056 (0.555–2.008)	0.869
Intrahepatic cholangiocarcinoma	6945	51	4.165 (1.255–13.828)	0.020	6894	50	0.700 (0.203–2.415)	0.572
Pancreatic adenocarcinoma	74,105	244	1.274 (0.963–1.684) *	0.090	73,837	243	1.479 (1.067–2.048) *	0.019
Small bowel adenocarcinoma	6033	117	2.511 (1.310–4.813)	0.006	6021	117	2.669 (1.369–5.206)	0.004
Colon adenocarcinoma								
Cecum	62,641	2006	0.955 (0.659–1.385)	0.809	60,367	1988	1.168 (0.790–1.727)	0.436
Appendix	6382	106	2.199 (0.854–5.666)	0.103	4789	105	2.008 (0.732–5.509)	0.176
Ascending colon	54,343	1967	0.443 (0.201–0.975)	0.043	52,517	1946	0.625 (0.284–1.374)	0.242
Transverse colon	26,637	901	0.987 (0.451–2.163)	0.974	25,753	886	1.049 (0.484–2.274)	0.904
Descending colon	16,721	526	0.548 (0.148–2.021)	0.366	16,084	518	0.591 (0.171–2.040)	0.406
Sigmoid colon	74,642	2567	0.834 (0.605–1.450)	0.268	71,723	2499	0.956 (0.682–1.339)	0.791
Rectal adenocarcinoma	73,835	2495	0.957 (0.883–1.037) *	0.285	73,654	2491	1.070 (0.942–1.216) *	0.298
Lung								
Adenocarcinoma	220,634	3046	1.163 (1.026–1.318) *	0.018	220,107	3045	1.076 (0.943–1.227) *	0.277
Squamous carcinoma	111,388	1669	1.232 (1.047–1.450)	0.012	110,971	1665	1.077 (0.877–1.322)	0.480
Small cell carcinoma	69,654	332	1.776 (1.016–3.106)	0.044	69,352	331	1.459 (1.013–2.103)	0.043
Large cell carcinoma	11,848	155	1.000 (0.638–1.568)	0.998	11,804	154	0.836 (0.520–1.344)	0.460
Renal cell carcinoma	42,776	1474	0.242 (0.120–0.489)	<0.001	42,630	1474	0.502 (0.200–1.261)	0.143
Prostate adenocarcinoma	582,190	30836	1.110 (1.041–1.183) *	0.001	579,521	30712	1.216 (1.138–1.300) *	<0.001
Urinary bladder transitional cell carcinoma	196,183	9969	1.477 (1.288–1.694) *	<0.001	195,777	9948	1.757 (1.516–2.037) *	<0.001
Thyroid								
Papillary thyroid carcinoma	104,715	2626	1.067 (0.987–1.152) *	0.102	104,680	2626	1.028 (0.948–1.114) *	0.504
Follicular thyroid carcinoma	6578	191	1.566 (1.155–2.123) *	0.004	6574	191	1.553 (1.134–2.127) *	0.006
Breast (women)								
Invasive ductal carcinoma	497,869	15236	/		497,312	15229	/	
Invasive lobular carcinoma	61,414	4450	1.373 (1.218–1.547)	<0.001	61,336	4444	1.108 (1.108–1.108)	<0.001
Ovarian epithelial cancer	49,690	932	0.841 (0.389–1.819)	0.660	49,636	931	0.744 (0.330–1.677)	0.475
Lymphoma								
Hodgkin lymphoma (nodal)	22,799	455	0.824 (0.642–1.057) *	0.128	22,799	455	0.872 (0.653–1.164) *	0.351
Non-Hodgkin lymphoma (nodal)	122,972	5652	1.230 (1.058–1.430)	0.007	122,972	5652	1.090 (0.948–1.254)	0.228
Melanoma (skin)	193,253	9865	1.425 (1.180–1.722) *	< 0.001	192,954	9855	1.109 (0.912–1.348) *	0.302

Model I: Fine and Gray models adjusting for age, sex, race, and tumor grade if available; Model II: Fine and Gray models adjusting for age, sex, race, tumor grade, AJCC-TNM stage, chemotherapy, and surgery if available. * Cox proportional hazards regression analyses were used. Sex was not adjusted in breast tumors (women) and prostate tumors (men). In lymphoma, tumor site was also adjusted in model II, while surgery was not adjusted. In small cell lung carcinoma, surgery was not adjusted in model II.

## Supplementary Materials

The following are available online at https://www.mdpi.com/1718-7729/28/1/35/s1. Figure S1: The cumulative incidences of death and secondary primary malignancy in colon adenocarcinoma divided by tumor sites before and after propensity score matching. Table S1: Codes for patient selection based on the International Classification of Diseases for Oncology, third edition (ICD-O-3), Table S2: Stratified analyses according to surgery.
