# Supplementary material for: The Impact of Radiotherapy on the Incidence of Secondary Malignancies: A Pan-Cancer Study in the US SEER Cancer Registries
Source: Curr Oncol. 2021 Jan 8;28(1):301–16. doi: 10.3390/curroncol28010035 (PMC7903277; doi:10.3390/curroncol28010035)

**The impact of radiotherapy on the incidence of secondary malignancies: a population-based study in the US SEER cancer registries**

**Wei Li*, MD, Haitao Xiao*, MD, Xuewen Xu, MD, Yange Zhang, MD**

**Author Affiliations:**

Department of Plastic and Burns Surgery, West China Hospital, Sichuan University, Chengdu, China.

| Supplementary Table 1. Codes for patient selection based on the International Classification of Diseases for Oncology, third edition (ICD-O-3). | |
| --- | --- |
| Tumor type | Code |
| Esophagus | Site code: C15. |
| Adenocarcinoma | Pathological code: 8140/3, 8141/3, 8144/3, 8200/3, 8210/3, 8211/3, 8244/3, 8255/3, 8260/3, 8261/3, 8263/3, 8310/3, 8323/3, 8480/3, 8481/3, 8560/3, 8570/3, 8574/3. |
| Squamous cell carcinoma | Pathological code: 8052/3, 8070-8076/3, 8083/3, 8084/3, 8094/3. |
| Gastric adenocarcinoma | Site code: C16; Pathological code: 8140/3, 8141/3, 8144/3, 8210/3, 8211/3, 8221/3, 8244/3, 8245/3, 8255/3, 8260-8263/3, 8290/3, 8310/3, 8323/3, 8410/3, 8441/3, 8480/3, 8481/3, 8503/3, 8560/3, 8570/3, 8574/3. |
| Hepatocellular carcinoma | Site code: C22.0; Pathological code: 8170-8175. |
| Intrahepatic cholangiocarcinoma | Site code: C22.1; Pathological code: 8160/3. |
| Pancreatic adenocarcinoma | Site code: C25; Pathological code: 8140/3,8141/3, 8144/3, 8210/3, 8211/3, 8244/3, 8245/3, 8255/3, 8260-8263/3, 8290/3, 8310/3, 8323/3, 8401/3, 8440/3, 8441/3, 8450/3, 8460/3, 8470/3, 8471/3, 8480/3, 8481/3, 8503/3, 8551/3, 8560/3, 8570/3, 8574/3. |
| Small bowel adenocarcinoma | Site code: C17; Pathological code: 8140/3, 8144/3, 8210/3, 8211/3, 8220/3, 8221/3, 8244/3, 8245/3, 8255/3, 8260-8263/3, 8310/3, 8441/3, 8470/3, 8480/3, 8481/3, 8560/3, 8572/3, 8574/3, 8576/3. |
| Colon adenocarcinoma | Site code: C18; Pathological code: 8140/3, 8141/3, 8143/3, 8144/3, 8147/3, 8210/3, 8211/3, 8213/3, 8220/3, 8221/3, 8244/3, 8245/3, 8255/3, 8260-8263/3, 8310/3, 8323/3, 8440/3, 8441/3, 8460/3, 8470-8472/3, 8480/3, 8481/3, 8551/3, 8560/3, 8570-8574/3, 8576/3. |
| Cecum | Site code: C18.0. |
| Appendix | Site code: C18.1. |
| Ascending colon | Site code: C18.2. |
| Transverse colon | Site code: C18.4. |
| Descending colon | Site code: C18.6. |
| Sigmoid colon | Site code: C18.7. |
| Rectal adenocarcinoma | Site code: C20.9; Pathological code: 8140/3, 8141/3, 8144/3, 8210/3, 8211/3, 8213/3, 8220/3, 8221/3, 8244/3, 8245/3, 8255/3, 8260-8263/3, 8310/3, 8323/3, 8441/3, 8480/3, 8481/3, 8560/3, 8570-8574/3, 8576/3. |
| Lung | Site code: C34. |
| Adenocarcinoma | Pathological code: 8140/3, 8141/3, 8144/3, 8147/3, 8200/3, 8211/3, 8244/3, 8245/3, 8250/3, 8251/3, 8255/3, 8260/3, 8263/3, 8272/3, 8290/3, 8310/3, 8323/3, 8333/3, 8401/3, 8410/3, 8441/3, 8460/3, 8470/3, 8471/3, 8480/3, 8481/3, 8503/3, 8525/3, 8551/3, 8560/3, 8570-8574/3, 8576/3. |
| Squamous carcinoma | Pathological code: 8052/3, 8070-8076/3, 8078/3, 8083/3, 8084/3, 8094/3. |
| Small cell carcinoma | Pathological code: 8041-8045. |
| Large cell carcinoma | Pathological code: 8011-8015. |
| Renal cell carcinoma | Site code: C64.9. Pathological code: 8312/3, 8316-8318/3. |
| Prostate adenocarcinoma | Site code: C61.9. Pathological code: 8312/3, 8316-8318/3. Pathological code: 8140/3, 8141/3, 8147/3, 8200/3, 8210/3, 8211/3, 8244/3, 8255/3, 8260/3, 8263/3, 8272/3, 8310/3, 8323/3, 8410/3, 8480/3, 8481/3, 8503/3, 8551/3, 8560/3, 8570-8574/3. |
| Urinary bladder carcinoma | Site code: C67. Pathological code: 8120/3, 8122/3, 8130/3, 8131/3. |
| Thyroid | Site code: C73.9. |
| Papillary thyroid carcinoma | Pathological code: 8050/3, 8260/3, 8340-8344/3. |
| Follicular thyroid carcinoma | Pathological code: 8330-8332/3, 8335/3. |
| Breast | Site code: C50. |
| Invasive ductal carcinoma | Pathological code: 8500/3. |
| Invasive lobular carcinoma | Pathological code: 8520/3. |
| Ovarian epithelial cancer | Site code: C56.9. Pathological code: 8441, 8442, 8460-8462, 9014, 8380-8383, 8470-8473, 8480-8482, 8050-8052, 8140, 8141, 8143, 8147, 8260-8263, 8310, 8313, 8440, 8450, 8451, 8560, 8562, 8570-8575, 8950, 8951, 8980-8982, 9000, 9015. |
| Lymphoma |  |
| Hodgkin lymphoma (nodal) | ICD-O-3/WHO 2008: Hodgkin-Nodal. |
| Non-Hodgkin lymphoma (nodal) | ICD-O-3/WHO 2008: Non-Hodgkin-Nodal. |
| Melanoma (skin) | ICD-O-3/WHO 2008: Melanoma of the skin. |

| Supplementary Table 2. Stratified analyses according to surgery. | | | |  |  |  |
| --- | --- | --- | --- | --- | --- | --- |
| Variables | Surgery | | | No surgery | | |
|  | Number | events | HR (95%CI) P value | Number | events | HR (95%CI) P value |
| Esophagus |  |  |  |  |  |  |
| Adenocarcinoma | 7725 | 286 | 0.776 (0.483-1.247) 0.295 | 14925 | 102 | 3.362 (1.243-9.095) 0.017 |
| Squamous cell carcinoma | 2126 | 89 | 0.975 (0.542-1.754) 0.932 | 10068 | 141 | 1.679 (0.715-3.947) 0.234 |
| Gastric adenocarcinoma | 20928 | 642 | 0.959 (0.784-1.174) 0.686 | 20595 | 76 | / |
| Hepatocellular carcinoma | 12955 | 422 | 0.595 (0.144-2.459) 0.473 | 35337 | 251 | 1.081 (0.650-1.798)* 0.764 |
| Intrahepatic cholangiocarcinoma | 1730 | 38 | 2.159 (0.918-5.074)* 0.078 | 5164 | 12 | / |
| Pancreatic adenocarcinoma | 10730 | 172 | 1.684 (1.075-2.640) 0.023 | 63107 | 71 | / |
| Small bowel adenocarcinoma | 3952 | 111 | 2.201 (1.185-4.091) 0.013 | 2069 | 6 | / |
| Colon adenocarcinoma | 254943 | 14675 | 0.998 (0.824-1.209)* 0.983 | 19503 | 169 | 5.376 (0.623-46.361)* 0.126 |
| Rectal adenocarcinoma | 58002 | 2336 | 1.401 (1.181-1.661) < 0.001 | 15652 | 155 | 2.354 (0.691-8.013) 0.171 |
| Lung |  |  |  |  |  |  |
| Adenocarcinoma | 71163 | 2398 | 1.255 (1.083-1.454)* 0.003 | 148944 | 647 | 0.840 (0.664, 1.063)* 0.148 |
| Squamous carcinoma | 31217 | 1173 | 0.837 (0.687-1.019) 0.077 | 79754 | 492 | 1.567 (1.027-2.391) 0.037 |
| Large cell carcinoma | 3337 | 118 | 0.786 (0.440-1.406) 0.418 | 8466 | 36 | 0.803 (0.380-1.696) 0.564 |
| Renal cell carcinoma | 27498 | 1338 | 0.891 (0.323-2.455)* 0.823 | 15132 | 136 | 1.340 (0.227-7.914) 0.747 |
| Prostate adenocarcinoma | 242082 | 10488 | 1.244 (1.159-1.335)* < 0.001 | 337439 | 20224 | 0.990 (0.791-1.238)* 0.927 |
| Urinary bladder transitional cell carcinoma | 183607 | 9516 | 1.766 (1.523-2.048)* < 0.001 | 12170 | 432 | / |
| Thyroid |  |  |  |  |  |  |
| Papillary thyroid carcinoma | 102593 | 2604 | 1.026 (0.947-1.113)* 0.530 | 2087 | 22 | 3.174 (0.680-14.820)* 0.142 |
| Follicular thyroid carcinoma | 6347 | 187 | 1.509 (1.101, 2.067)* 0.010 | 227 | 4 | / |
| Breast |  |  |  |  |  |  |
| Invasive ductal carcinoma | 467150 | 14900 | / | 30162 | 329 | / |
| Invasive lobular carcinoma | 56639 | 4218 | 1.234 (1.021-1.492) < 0.001 | 4697 | 226 | 0.535 (0.195-1.470) 0.225 |
| Ovarian epithelial cancer | 40897 | 897 | 0.719 (0.329-1.569) 0.407 | 8757 | 34 | / |
| Melanoma (skin) | 182719 | 10803 | 1.179 (0.958-1.452)* 0.121 | 11478 | 280 | 1.011 (0.570-1.791)* 0.971 |
| Fine & Gray models adjusting for age, sex, race, tumor grade, AJCC-TNM stage and chemotherapy when the data is available. *Cox proportional hazards regression analyses were used. | | | | | | |
|  |  |  |  |  |  |  |
|  |  |  |  |  |  |  |

| Supplementary Table 3. Stratified analyses according to sex. | | | | | | |
| --- | --- | --- | --- | --- | --- | --- |
| Variables | Male | | | Female | | |
|  | Number | events | HR (95%CI) P value | Number | events | HR (95%CI) P value |
| Esophagus |  |  |  |  |  |  |
| Adenocarcinoma | 19659 | 45 | 1.593 (1.189-2.133) 0.002 | 3087 | 345 | 2.363 (1.112-5.020) 0.025 |
| Squamous cell carcinoma | 7980 | 134 | 1.366 (0.968-1.928) 0.076 | 4252 | 96 | 1.514 (1.015-2.259) 0.042 |
| Gastric adenocarcinoma | 28361 | 531 | 1.441 (1.108-1.873) 0.006 | 13282 | 188 | 1.897 (1.230-2.927) 0.004 |
| Hepatocellular carcinoma | 46445 | 757 | 1.976 (0.621-6.284)* 0.249 | 13511 | 230 | 2.059 (0.654-6.485) 0.217 |
| Intrahepatic cholangiocarcinoma | 3553 | 23 | 1.025 (0.754-1.394)* 0.874 | 3426 | 28 | 1.324 (0.992-1.766)* 0.056 |
| Pancreatic adenocarcinoma | 38261 | 134 | 1.010 (0.957-1.065) 0.729 | 35844 | 110 | 0.964 (0.911-1.021) 0.214 |
| Small bowel adenocarcinoma | 3293 | 72 | 4.398 (1.337-14.465) 0.015 | 2741 | 45 | 7.057 (1.898-26.240) 0.004 |
| Colon adenocarcinoma | 135885 | 8429 | 1.156 (1.092-1.223)* < 0.001 | 138561 | 6415 | 1.060 (0.995-1.129)* 0.071 |
| Rectal adenocarcinoma | 44289 | 1527 | 1.044 (0.890-1.224)* 0.599 | 29546 | 968 | 1.374 (1.121-1.685)* 0.002 |
| Lung |  |  |  |  |  |  |
| Adenocarcinoma | 107145 | 1524 | 0.839 (0.649-1.085)* 0.181 | 113489 | 1522 | 1.074 (0.902-1.280)* 0.424 |
| Squamous carcinoma | 69995 | 1118 | 0.950 (0.765-1.180) 0.642 | 41393 | 551 | 0.697 (0.455-1.069) 0.098 |
| Large cell carcinoma | 6792 | 81 | 0.676 (0.330-1.383) 0.283 | 5056 | 74 | 0.962 (0.490-1.892) 0.911 |
| Renal cell carcinoma | 26398 | 482 | 0.560 (0.234-1.341)* 0.193 | 16378 | 992 | 0.229 (0.034-1.564)* 0.133 |
| Urinary bladder transitional cell carcinoma | 149048 | 8119 | 1.398 (1.185-1.649)* < 0.001 | 47135 | 1850 | 1.006 (0.723-1.401)* 0.972 |
| Thyroid |  |  |  |  |  |  |
| Papillary thyroid carcinoma | 80636 | 1855 | 1.012 (0.871-1.175)* 0.880 | 24079 | 771 | 1.032 (0.938-1.135)* 0.523 |
| Follicular thyroid carcinoma | 4637 | 85 | 1.568 (0.974-2.525)* 0.064 | 1941 | 106 | 1.607 (1.065-2.426)* 0.024 |
| Melanoma (skin) | 113168 | 6453 | 1.243 (1.181-1.309)* < 0.001 | 80085 | 3412 | 1.337 (1.228-1.457)* < 0.001 |
| Fine & Gray model adjusting for age, race, tumor grade, AJCC-TNM stage, surgery and chemotherapy when the data is available. *Cox proportional hazards regression analyses were used. | | | | | | |
|  |  |  |  |  |  |  |

Supplementary Fig. 1 The cumulative incidences of death and secondary primary malignancy in colon adenocarcinoma divided by tumor sites before and after propensity score matching.


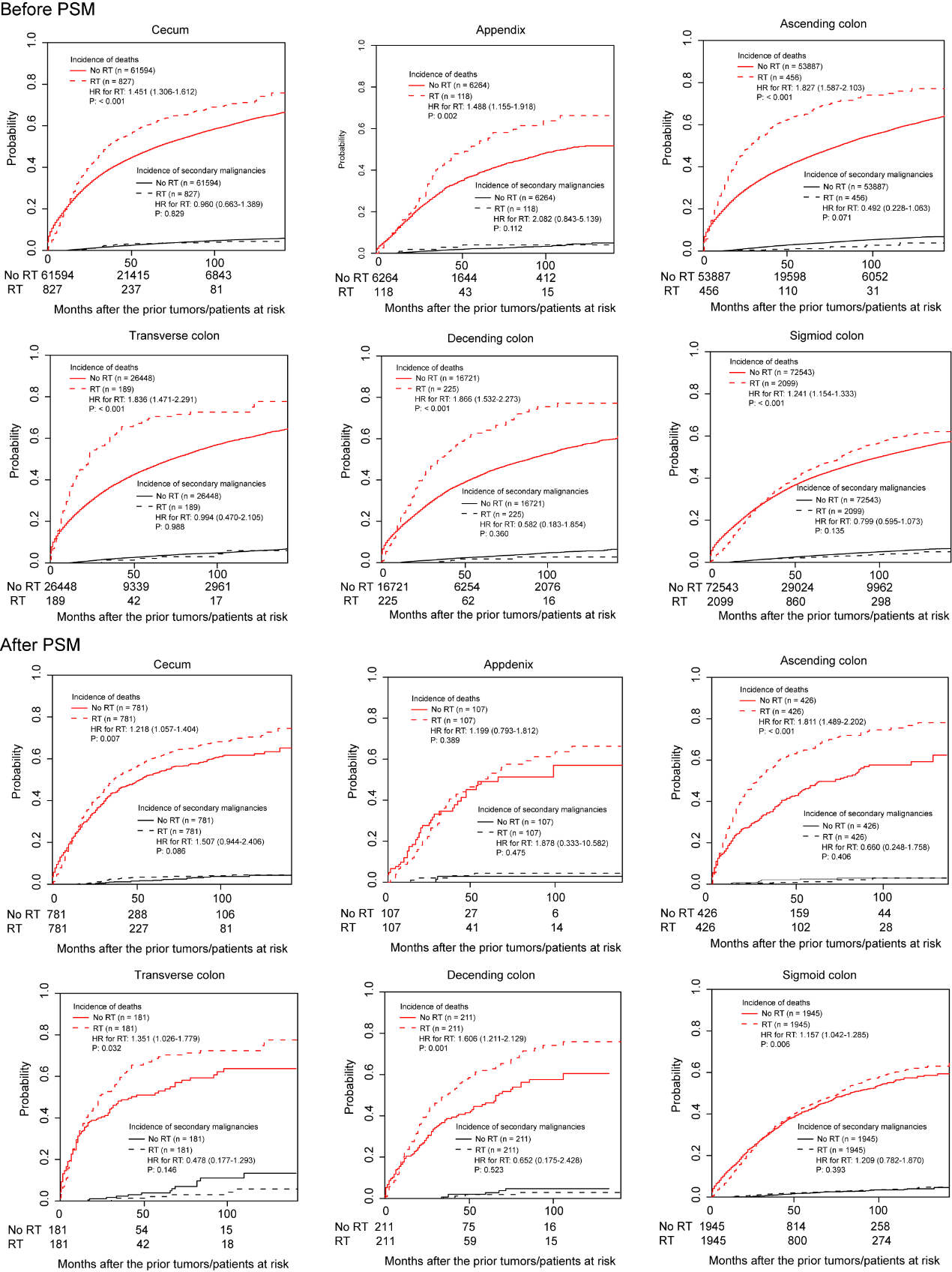

Supplement: Supplementary file 1 [file curroncol-28-00035-s001.zip › curroncol-1013102supp.docx]
